# Non-Contiguous Cervical and Thoracic Spinal Cord Injuries After Severe Polytrauma: A Case Report

**DOI:** 10.3390/reports9040330

**Published:** 2026-09-28

**Authors:** Fernando Martins Braga, Elena Tello Santamaria, Margarita Vallès, Hatice Kumru

**Affiliations:** 1 Institut Guttmann Hospital de Neurorehabilitació, 08916 Badalona, Catalonia, Spain; mvalles@guttmann.com (M.V.);; 2 Institut de Neurociències (INc), Universitat Autònoma de Barcelona (UAB), 08193 Bellaterra, Catalonia, Spain; 3 Hospital Universitari Son Espases, 07120 Palma de Mallorca, Illes Balears, Spain; elena.tello@ssib.es

**Keywords:** non-contiguous spinal cord injury, polytrauma, traumatic brain injury, ISNCSCI, case report

## Abstract

**Background and Clinical Significance:** Non-contiguous traumatic spinal cord injury (SCI) can be difficult to recognise and classify in severe polytrauma, especially when traumatic brain injury (TBI) and limb injuries limit neurological examination. **Case Presentation:** A 17-year-old male was involved in a motorcycle accident resulting in severe polytrauma with TBI. Brain magnetic resonance imaging (MRI) showed a grade III diffuse axonal injury. Trauma computed tomography (CT) identified C2 and T3–T5 vertebral fractures. Spinal MRI revealed a non-haemorrhagic C3–C6 contusive myelopathy, a T4 spinal cord contusion, and a thoracic posterior epidural haematoma. He underwent posterior T2–T7 fixation. Acute neurological examination was limited by impaired consciousness and associated injuries. At four months, the International Standards for Neurological Classification of Spinal Cord Injury (ISNCSCI) assessment showed a neurological level of C2, and a total motor score of 55. Electrodiagnostic testing showed active bilateral C5–T1 denervation. At seven months and one year, the total motor score increased to 63 and 82, respectively, accompanied by substantial functional improvement. **Conclusions:** In severe polytrauma with TBI, non-contiguous SCI should be considered when neurological examination is unreliable. In non-contiguous SCI, clinical assessment should integrate neurological examination, including quantitative motor and sensory scores, with imaging and neurophysiological studies, cognitive status, associated injuries, and functional outcomes.

## 1. Introduction and Clinical Significance

Non-contiguous spinal injuries may present a diagnostic challenge in individuals with severe polytrauma [1,2]. They can be difficult to recognise during the initial assessment [1,3], especially when resuscitation and the treatment of life-threatening associated injuries take priority [2]. Concomitant traumatic brain injury (TBI) can further interfere with diagnosis, as altered consciousness, sedation, or intubation can delay a reliable neurological examination and, consequently, classification of the spinal cord injuries (SCI) [1,4].

For this reason, spinal magnetic resonance imaging (MRI) has been proposed during the acute evaluation of polytrauma when traumatic SCI cannot be reliably excluded. Computed tomography (CT) is best suited to identifying vertebral injuries, but MRI may be required to characterise spinal cord involvement [2,5,6]. However, identifying a non-contiguous SCI does not resolve the problem of neurological classification [7].

The International Standards for Neurological Classification of Spinal Cord Injury (ISNCSCI) provide the most widely used standardised framework for determining the neurological level and classifying the severity of SCI using the American Spinal Injury Association Impairment Scale (AIS) [7,8]. Because this classification is derived from sensory and motor examination findings, abnormalities caused by coexisting non-SCI conditions (e.g., brachial plexus or other peripheral nerve injuries) can influence the result and must therefore be recognised [7]. The 2019 ISNCSCI revision introduced a taxonomy for documenting non-SCI conditions [9]. Abnormal sensory or motor scores affected by such conditions are recorded as examined and marked with an asterisk (*), with explanatory comments specifying their handling during classification [10]. Published case examples illustrate how these rules apply when concomitant non-SCI conditions affect sensory or motor examination findings [11].

In individuals with non-contiguous SCI, distal involvement can obscure the degree of motor and sensory preservation attributable to a more proximal injury. A neurologically complete distal injury results in no sacral sparing on the combined neurological examination, making it impossible to determine whether the proximal injury would otherwise be neurologically incomplete. For this reason, a single ISNCSCI classification may not adequately describe such cases [7].

This case report describes non-contiguous cervical and thoracic traumatic SCI in an adolescent with severe polytrauma and TBI who was admitted for specialised neurorehabilitation. The case illustrates the challenges of neurological assessment and classification when non-contiguous SCI coexists with severe TBI and additional peripheral neurological injuries, with implications for prognostication, clinical management, and rehabilitation planning. This report was prepared in accordance with the CARE guidelines.

## 2. Case Presentation

A previously healthy 17-year-old male was involved in a motorcycle accident resulting in severe polytrauma. The initial assessment was consistent with a severe TBI with a Glasgow Coma Scale score of 3, and the patient therefore required intubation and mechanical ventilation. Cranial CT showed bilateral frontal fractures with associated intraparenchymal haemorrhages, and brain MRI demonstrated a grade III diffuse axonal injury. Due to poor intracranial pressure control, an external ventricular drain was placed, followed by ventriculoperitoneal shunt insertion approximately six weeks after injury.

Initial trauma CT identified a right lateral-mass fracture of C2 without osseous canal compromise and fractures of T3–T5. Spinal MRI was initially postponed because of haemodynamic instability and was performed six days after the injury. It demonstrated a T4 burst fracture with slight posterior wall retropulsion, a posterior epidural haematoma in the mid and lower thoracic spine, a non-haemorrhagic contusive myelopathy extending from C3 to C6 without a corresponding vertebral injury at those levels, considered suggestive of spinal cord injury without radiographic abnormality (SCIWORA), and a separate T4 spinal cord contusion related to the burst fracture (Figure 1). The C2 fracture was treated conservatively. Eighteen days after the injury, the patient underwent posterior T2–T7 fixation with pedicle screws due to the unstable thoracic fractures. Follow-up cervicothoracic MRI approximately two and a half months after injury showed persistent cervical spinal cord swelling with central oedema extending from C2 to C6 and post-contusive myelomalacic foci at T4–T5; the T2–T7 instrumentation maintained satisfactory alignment with mild residual kyphotic angulation.

Associated limb injuries included bilateral forearm and wrist fractures requiring surgical fixation, an open left tibial plateau fracture requiring fixation, and traumatic distal amputations of the right middle and ring fingers. During the acute phase, severely impaired consciousness, inconsistent sensory responses, limb injuries requiring immobilisation, and the presence of non-contiguous SCI limited the reliability and interpretation of the neurological examination. For this reason, the amount of neurological recovery attributable specifically to thoracic fixation could not be determined. Electrodiagnostic testing performed three months after injury demonstrated active bilateral C5–T1 denervation with markedly impaired voluntary recruitment, while upper-limb sensory nerve conduction was normal (Table 1).

On admission to neurorehabilitation, approximately four months after injury, the patient was conscious, oriented, cooperative, and able to communicate verbally, although short-term memory deficits persisted. The first ISNCSCI assessment performed at our unit identified a neurological level of C2, with a total motor score of 55. Sacral examination showed altered light touch and pin prick at S4–5 bilaterally; deep anal pressure (DAP) and voluntary anal contraction (VAC) were present. Neurological examination showed asymmetric tetraparesis, with the most pronounced weakness affecting the distal left lower limb. Additional clinical features included bilateral lower-limb spasticity with hyperreflexia and clonus, neurogenic bladder and bowel dysfunction, and neuropathic pain. He was unable to stand or walk and remained dependent for transfers and personal care.

The inpatient neurorehabilitation programme included physiotherapy focused on range of motion, strengthening and gait retraining; occupational therapy addressing activities of daily living, transfers, hand function and wheelchair skills; neuropsychological treatment targeting cognitive deficits; and psychological treatment focused on adjustment to disability and stress management, with support for the patient and his family.

At the follow-up assessment, seven months after injury, the total motor score had improved from 55 to 63. The marked distal weakness of the left lower limb persisted. Electrodiagnostic testing at eight months identified a left common fibular neuropathy at the knee level, near the tibial plateau fracture, which partly accounted for the marked left–right asymmetry in distal lower-limb weakness. By the end of inpatient rehabilitation, approximately one year after injury, the final ISNCSCI assessment showed a persistent neurological level of C2, with a total motor score of 82. Sacral examination showed normal light touch and altered pin prick at S4–5 bilaterally; DAP and VAC remained present. Accordingly, the left L4 and L5 motor scores at the 12-month ISNCSCI examination were annotated as affected by a non-SCI condition, while the original examined scores were retained (Figure 2).

Functional independence also improved, with the Shah-modified Barthel Index (0–100) increasing from 5 (rehabilitation admission) to 20 (seven months) and 55 (final assessment, six days before discharge). The Functional Independence Measure (FIM) total score (18–126) increased from 44 (rehabilitation admission) to 99 (discharge), with the motor sub-score increasing from 14 to 69 and the cognitive sub-score remaining at 30. At discharge, the patient‘s independence had improved in several domains, including feeding, personal hygiene, bathing/showering, transfers, toileting, dressing, and level-ground mobility, although occasional assistance was still required. He remained unable to manage stairs independently. During late rehabilitation, he could walk short distances with a walker and a lower-limb orthosis. Over longer distances, he used an electric wheelchair to move independently.

Bladder function had improved to continence, with spontaneous micturition and no significant residual volume. Bowel management consisted of evacuation every 48 h using a sodium citrate rectal micro-enema and oral magnesium hydroxide. Neuropsychological follow-up showed improvement in memory and executive control, although mild deficits persisted. Detailed ISNCSCI examination findings at 4 and 12 months are presented in Figure 2. The clinical course is summarised in Figure 3.

## 3. Discussion

The present case illustrates the challenge of anatomically attributing neurological deficits over time when non-contiguous cervical and thoracic SCI coexist with severe TBI and superimposed peripheral neurological injury. In the acute phase, severely impaired consciousness, inconsistent sensory responses, limb injuries requiring immobilisation, and the need to prioritise life-saving interventions can limit the feasibility of a complete ISNCSCI assessment [13]. In this case, the vertebral fractures identified on initial trauma CT raised suspicion of spinal cord involvement, but a full-spine MRI was required to establish the non-contiguous cervical and thoracic spinal cord lesions. The absence of a corresponding C3–C6 vertebral injury suggests that the cervical spinal cord lesion resulted from transient traumatic deformation rather than persistent osseous compression, consistent with the SCIWORA interpretation of the acute MRI.

In the broader context of spinal trauma, previous studies have highlighted the risk of additional spinal injuries being overlooked. In a series of 484 individuals with spinal trauma, Kanna et al. identified non-contiguous spinal injuries in 86 patients (17.8%), with cervical–thoracic involvement representing the most frequent pattern (29.1%). Whole-spine MRI detected 24 clinically unsuspected secondary spinal injuries, five of which were unstable and required surgical stabilisation [14]. Turning specifically to spinal cord involvement, in an earlier series of 71 patients with acute SCI, Silberstein and McLean identified seven patients (10%) with non-contiguous SCI. In all seven, the clinical findings were not fully explained by conventional radiographs or CT, and MRI demonstrated additional spinal cord abnormalities that helped account for the neurological presentation [15].

The limitations of assigning a single neurological classification in non-contiguous SCI are already recognised in the ISNCSCI literature. Kirshblum et al. addressed this problem in an illustrative scenario involving an incomplete cervical SCI and a complete T6 SCI. Because the complete distal injury resulted in combined examination findings consistent with neurological completeness, even though the proximal cervical injury was incomplete, they recommended documenting the limitation in the ISNCSCI worksheet comment box rather than assigning a single AIS grade [7]. This distinction is clinically relevant because neurological level and injury severity, as reflected by AIS grade, are important prognostic variables [16]; uncertainty regarding the severity of the proximal injury may therefore affect expectations regarding neurological recovery and rehabilitation planning.

In the present case, as consciousness, cooperation, and the conditions for limb examination improved during rehabilitation, ISNCSCI assessment became more reliable. The clinical designation remained AIS D, while the total motor score increased from 55 at four months to 63 at seven months and 82 at one year, accompanied by substantial functional gains. Krishna et al. note that AIS is an ordinal classification and that functional outcomes correlate more closely with quantitative motor and sensory scores than with AIS grade [16]. ASIA guidance recommends recording the most rostral neurological levels without assigning a single AIS grade in non-contiguous multiple SCI [7]. In our patient, preserved sacral sensation, DAP and VAC established motor-incomplete status, and the combined motor examination met the numerical criteria for AIS D. This designation was used solely for clinical communication, as the combined examination does not allow the AIS grade of the thoracic injury to be determined independently of the effects of the cervical lesion and peripheral nerve injury.

Neurophysiological assessment can complement the neurological examination when weakness is difficult to interpret clinically [17]. In the present case, the first study showed active bilateral C5–T1 denervation with normal upper-limb sensory nerve conduction, although the exact localisation between anterior horn and motor root could not be determined. The later study demonstrated a left common fibular neuropathy at the knee (fracture) level, with possible sural involvement, confirming a peripheral contribution to distal left-lower-limb weakness. The C3–C6 cervical spinal cord lesion, interpreted as SCIWORA, could account for the bilateral lower-limb upper-motor-neuron signs and contribute to the tetraparesis, but the relative contributions of the cervical spinal cord injury and C5–T1 lower-motor-neuron abnormalities to upper-limb weakness could not be distinguished. Severe TBI limited the reliability of the acute neurological examination and influenced the early functional presentation. At four months, no neurological deficit could be assigned specifically to the T4 lesion, because deficits below T4 could also be explained by the cervical SCI.

The functional gains observed during rehabilitation likely reflected interacting neurological and non-neurological factors, including improvement in cognitive status, reduced restrictions from associated injuries, rehabilitation training, and partial motor and sensory recovery. Scivoletto et al. found that, in some subgroups, patients with SCI and multiple associated injuries had poorer functional status at rehabilitation admission but not at discharge, while neurological level and completeness remained the main determinants of outcome [18]. These findings suggest that early functional status may lead clinicians to underestimate rehabilitation potential when associated injuries still restrict performance, supporting reassessment of functional goals as these restrictions resolve.

An important strength of this report is the availability of serial standardised neurological and functional assessments throughout a specialised inpatient rehabilitation programme. A limitation is the absence of a reliable acute ISNCSCI assessment, as the severe TBI and associated injuries rendered a reliable neurological examination impossible. In severe polytrauma, ISNCSCI remains an essential tool for standardised neurological follow-up, but it should be interpreted together with imaging findings, neurophysiological data, cognitive status, associated injuries, and functional outcomes.

## 4. Conclusions

Non-contiguous SCI should be considered after severe polytrauma, particularly when TBI or associated injuries prevent a reliable neurological examination. Although CT can demonstrate vertebral injuries, full-spine MRI may be required to define the distribution of spinal cord involvement. When non-contiguous SCI coexists with other central or peripheral neurological injuries, neurological assessment should integrate ISNCSCI findings with imaging, neurophysiological data, cognitive status, associated injuries, and functional outcomes to refine prognostic interpretation and guide rehabilitation planning in complex cases.

## Figures and Tables

**Figure 1:**
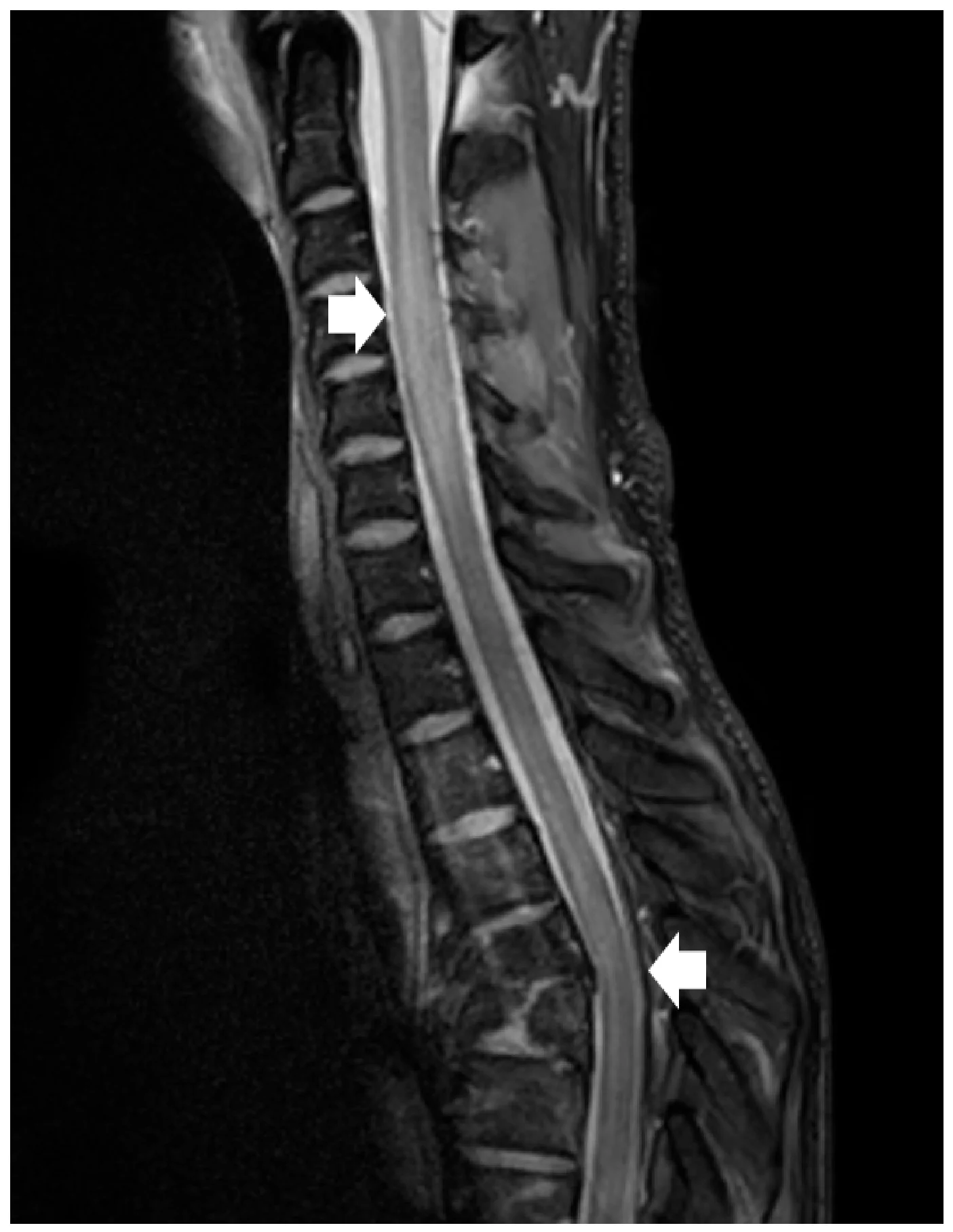
Sagittal T2-weighted spinal magnetic resonance imaging (MRI) demonstrating two non-contiguous cervical and thoracic spinal cord lesions. Upper arrow: cervical spinal cord signal abnormality extending from C3 to C6. Lower arrow: a separate thoracic spinal cord lesion at T4.

**Figure 2:**
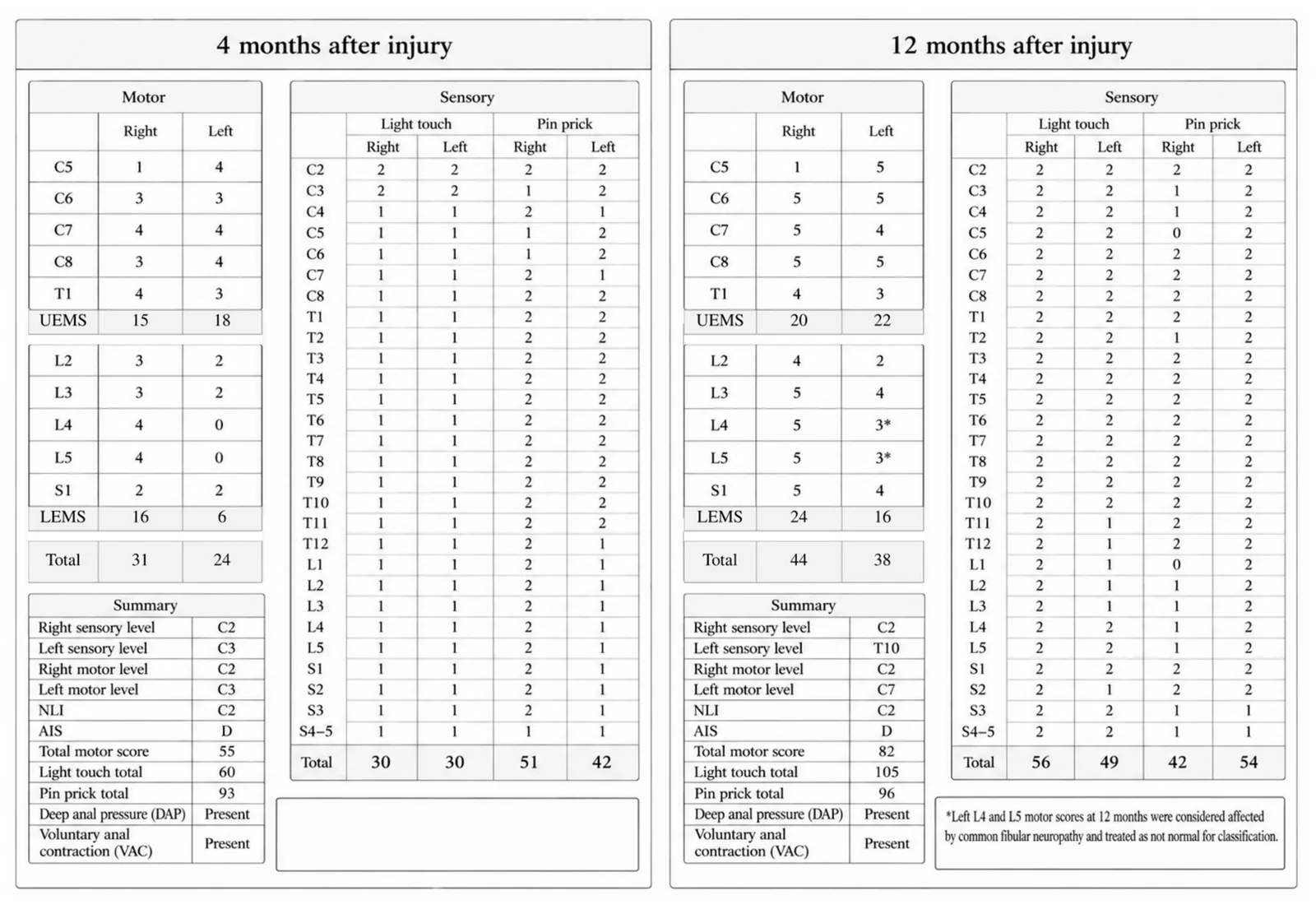
International Standards for Neurological Classification of Spinal Cord Injury (ISNCSCI) examination findings at 4 and 12 months after injury according to the 2026 ISNCSCI standards [12]. A single AIS grade D is shown only for clinical communication purposes and should not be interpreted as a definitive classification of the non-contiguous injuries or of either lesion individually. AIS: American Spinal Injury Association Impairment Scale; LEMS: Lower Extremity Motor Score; NLI: neurological level of injury; UEMS: Upper Extremity Motor Score.

**Figure 3:**
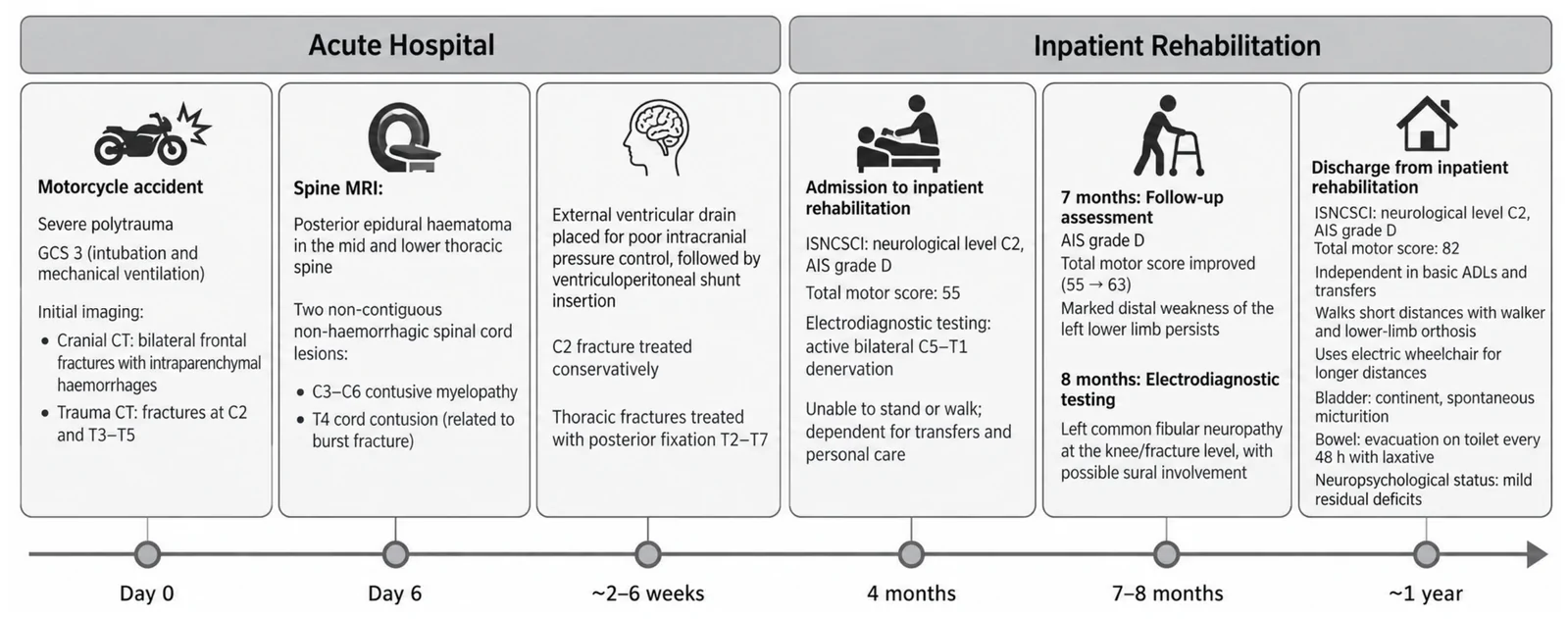
Timeline of the clinical course. The AIS designation is used as explained in Figure 2 legend. ADLs: activities of daily living; AIS: American Spinal Injury Association Impairment Scale; CT: computed tomography; EMG: electromyography; GCS: Glasgow Coma Scale; ISNCSCI: International Standards for Neurological Classification of Spinal Cord Injury; MRI: magnetic resonance imaging.

**Table 1:** Summary of electrodiagnostic findings and their neurological interpretation.

Parameter	3 Months—Upper Limbs	8 Months—Lower Limbs
Conduction studies	Median, ulnar and radial sensory NCS normal bilaterally. Central motor conduction to C8 within normal limits.	Right sural normal. Left common fibular sensory response absent and sural response markedly reduced. Left tibial motor response to AH normal; response recorded from soleus reduced.
Muscles examined by needle EMG	Bilateral deltoid, biceps, triceps, extensor carpi radialis, flexor carpi radialis, FDI and APB.	TA, soleus and EDB (left side).
Needle EMG findings	Abundant spontaneous activity bilaterally in all muscles. No voluntary activation in right deltoid/biceps; only 1–2 motor units recruited in the remaining muscles. Motor-unit morphology was normal.	Abundant spontaneous activity in TA and EDB. No voluntary motor-unit recruitment in TA and EDB.
Interpretation	Preganglionic involvement: Active bilateral C5–T1 denervation; the exact localisation between the anterior horn and the motor root could not be determined.	Left common fibular neuropathy at the knee (fracture) level.

AH: abductor hallucis; APB: abductor pollicis brevis; EDB: extensor digitorum brevis; EMG: electromyography; FDI: first dorsal interosseous; NCS: nerve conduction studies; TA: tibialis anterior.

## Data Availability

The clinical data supporting this case report are not publicly available due to patient privacy and confidentiality re-strictions. Relevant de-identified information is included within the article.

## Abbreviations

The following abbreviations are used in this manuscript:

CT	Computed tomography
DAP	Deep anal pressure
EMG	Electromyography
FDI	First dorsal interosseous
FIM	Functional Independence Measure
GCS	Glasgow Coma Scale
ISNCSCI	International Standards for Neurological Classification of Spinal Cord Injury
MRI	Magnetic resonance imaging
NCS	Nerve conduction studies
NLI	Neurological level of injury
SCI	Spinal cord injury
SCIWORA	Spinal cord injury without radiographic abnormality
TA	Tibialis anterior
TBI	Traumatic brain injury
VAC	Voluntary anal contraction
